# Neurocysticercosis as a first presentation of tonic-clonic seizures: a case report

**DOI:** 10.1186/1757-1626-1-104

**Published:** 2008-08-18

**Authors:** Matthew J Booker, Catherine Snelson, Louise Dodd

**Affiliations:** 1 Intensive Care Department, City Hospital, Birmingham, UK

## Abstract

We report the case of a 28 year-old immigrant Asian man from the Punjab region with a first presentation of seizures. This patient had no significant past medical history, but suffered several headaches in the preceding week and was pyrexial on presentation. A CT scan of his head showed a single area of subcortical low attenuation initially suggesting ischaemia. A lumbar puncture and CSF examination was unremarkable. Further investigation revealed discrete calcified gluteal lesions on pelvic X-ray, and serum immunology positive for cysticercosis. The diagnosis of neurocysticercosis was made, and the patient improved on dexamethasone and a short course of vermicide, to be discharged a week later. With increasing global migration, the prevalence of neurological parasitic infections seen in the UK is likely to rise. This case highlights the importance of careful interpretation of non-specific head CTs in the context of first presentation of seizures in a susceptible population.

## Background

Cysticercosis is the commonest parasitic infestation of the central nervous system worldwide. It is caused by the ingestion of the eggs or larvae of the tapeworm *Taenia solium*, found in faecally contaminated water and undercooked pork, affecting the gut initially and spreading haematogenously [1]. Sufferers often experience a long asymptomatic period, and can present with a variety of neurological manifestations, including focal neurological deficits, migraines, visual hallucinations and seizures [2]. The diagnosis of cysticercosis is often only made coincidentally on post-mortem examination. Extra-neurological manifestations include ocular deposition and skeletal muscle nodules. As such, careful fundoscopy and plain film radiology are mandatory for all in whom the diagnosis is suspected. A single short course of the vermicidal albendazole is usually sufficient to clear an infestation. The patient will need repeat imaging after several months to ensure complete eradication. Even having achieved this, long-term neurological sequelae are not uncommon.

## Case presentation

The patient, a 28 year-old Asian man, was admitted via ambulance to the Accident & Emergency department of City Hospital, Birmingham UK, following three successive witnessed tonic-clonic seizures. Each seizure lasted approximately 6 minutes, being separated by several minutes of incomplete recovery. The patient had complained to relatives of minor headaches during the preceding week, settling with simple analgesia. He was otherwise fit and well, on no medication, with no significant past medical history of note and no history of head trauma. He moved to the UK from the Punjab region 10 years previously, and has not been abroad since.

On presentation to the Emergency Department he had a GCS of 6, a temperature of 38.5°C, and subsequently fitted again. His right pupil was comparatively slightly dilated, but fundoscopy appeared normal. He was intubated and ventilated in the Emergency Department for transfer to the CT scanner. Immediately following the head CT scan a lumbar puncture was performed, the results of which were within normal limits with no organisms seen by Gram staining.

**Figure 1:**
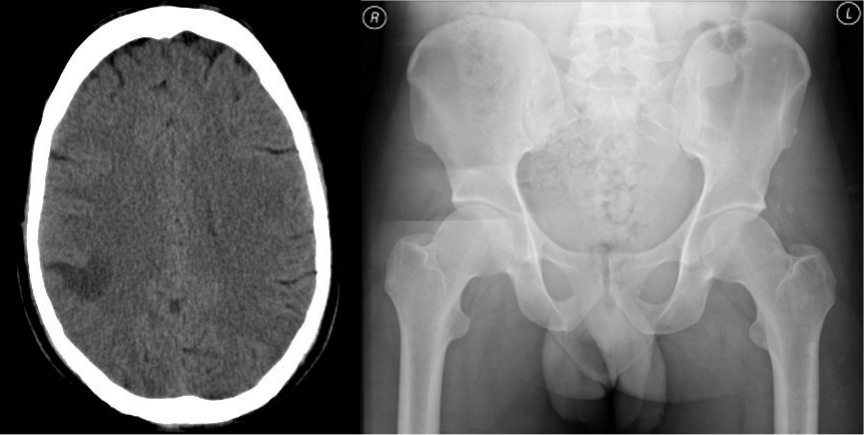
**Head CT scan at presentation and pelvic plain film x-ray**. Left: Head CT showing an area of subcortical reduced attenuation that could be mistaken for ischaemia. Right: Pelvic x-ray demonstrating several small, discrete, calcified lesions in the left gluteal muscles.

The head CT scan showed a single low attenuation area extending from the right lateral ventricle to the cortex (fig 1), but no other indications of raised intracranial pressure. Whilst it was initially thought in keeping with ischaemia, following neuroradiological review the diagnosis of cerebral cysticercosis was made. A plain pelvic X-Ray demonstrated multiple small calcific densities projecting into the soft tissues of the left gluteus (fig 1), and a subsequent serum cysticercosis immunoblot was positive. He was treated with albendazole and dexamethasone, his seizures settled and he was discharged from hospital 7 days later.

## Discussion

In our case, the seizures were most likely due to cerebral oedema rather than the parasite itself, as evidenced by their settling with dexamethasone. Although not a feature of this case, cysticercosis antibodies and antigens can be detected in the CSF when it is the cause of meningitis. In the developing world the vascular complications of neurocysticercosis are an important cause of haemorrhagic and ischaemic stroke.

The 'classic' CT appearance of neurocysticercosis is a single enhancing ring lesion, with or without scolex. This is not always seen however, particularly in Asian patients [3]. Indeed, as this case highlights, a less well-defined marginally enhancing subcortical lesion could be mistaken for an area of ischaemia.

This case highlights that, with ever increasing worldwide migration, the diagnosis of neurocysticercosis is likely to become more common [4], and should be considered in patients presenting with seizures in whom social history is commensurate and initial imaging non-diagnostic.

## Competing interests

The authors declare that they have no competing interests.

## Authors' contributions

MB principally authored the manuscript, and undertook the literature review. CS and LD collected primary data and were major contributors to the manuscript. All authors have read and approved the final manuscript.

## Consent

Written informed consent was obtained from the patient for the publication of this case report and accompanying images. A copy of the written consent is available for review by the Editor-in-Chief of this journal.

## References

[B1] Carpio A (2002). Neurocysticercosis: an update. Lancet Infectious Diseases.

[B2] Kraft R (2007). Cysticercosis; an emerging parasitic disease. American Family Physician.

[B3] Garg RK (2004). Diagnostic criteria for neurocysticercosis: some modifications are needed for Indian patients. Neurology India.

[B4] Pal DK, Carpio A, Sander JW (2000). Neurocysticercosis and epilepsy in developing countries. J Neurol Neurosurg Psychiatry.

